# The coat protein p25 from maize chlorotic mottle virus involved in symptom development and systemic movement of tobacco mosaic virus hybrids

**DOI:** 10.3389/fmicb.2022.951479

**Published:** 2022-08-05

**Authors:** Chao Zhang, Di Wang, Weimin Li, Baolong Zhang, Gamal M. Abdel-Fattah Ouf, Xiaofeng Su, Jun Li

**Affiliations:** ^1^State Key Laboratory of North China Crop Improvement and Regulation, College of Life Sciences, Hebei Agricultural University, Baoding, China; ^2^Center for Advanced Measurement Science, National Institute of Metrology, Beijing, China; ^3^Biotechnology Research Institute, Chinese Academy of Agricultural Sciences, Beijing, China; ^4^Excellence and Innovation Center, Jiangsu Academy of Agricultural Sciences, Nanjing, China; ^5^Department of Botany and Applied Microbiology, Faculty of Science, Mansoura University, Mansoura, Egypt

**Keywords:** MCMV, p25, pathogenicity, systemic movement, *HSP90*

## Abstract

Viral coat protein (CP) has numerous critical functions in plant infection, but little is known about p25, the CP of maize chlorotic mottle virus (MCMV; *Machlomovirus*), which causes severe yield losses in maize worldwide. Here, we investigated the roles of p25 in pathogenicity and systemic movement, as well as potential interactions with host plants, using a hybrid tobacco mosaic virus (TMV)-based expression system. Highly conserved protein p25 is predicted to contain a membrane-anchored nuclear localization signal (NLS) sequence and an extracellular sequence. In transgenic *Nicotiana benthamiana* plants containing the movement protein (MP) of TMV (TMV-MP), p25 induced severe symptoms, including dwarf and foliar necrosis, and was detected in inoculated and non-inoculated leaves. After the deletion of NLS from nuclear-located p25, the protein was found throughout the host cell, and plant stunting and starch granule deformity were reduced. Systemic movement and pathogenicity were significantly impaired when the C-terminal regions of p25 were absent. Using virus-induced gene silencing (VIGS), the transcript level of heat shock protein *HSP90* was distinctly lower in host plants in association with the absence of leaf necrosis induced by TMV-p25. Our results revealed crucial roles for MCMV p25 in viral pathogenicity, long-distance movement, and interactions with *N. benthamiana*.

## Introduction

Maize chlorotic mottle virus (MCMV; genus *Machlomovirus*, family Tombusviridae), a positive-sense, single-stranded RNA maize virus (Nutter et al., 1989), is an exclusive pathogen of maize around the world. First reported in Peru in 1974, then in the United States in 1976, this virus is now widespread (Niblett and Claflin, 1978; Lommel et al., 1991; Mahuku et al., 2015). It was first detected in the Yunnan province of China in 2009 (Xie et al., 2011). The only natural host of MCMV is maize, and its host range is limited to several gramineous plants. Transmitted primarily by seed, corn residues, thrips, and leaf beetles, the virus is listed for import inspection and quarantine because of the tremendous threat it poses to maize-based food security worldwide (Bockelman, 1982; Jensen, 1991; Cabanas et al., 2013; Wang et al., 2014).

In general, MCMV infection can cause slight chlorosis and mottling, yellow striping, and dwarfing. It causes severe corn lethal necrosis disease and serious yield losses when present in maize in a mixed infection with various maize-infecting potyviruses and even in single infection (Goldberg and Brakke, 1987; Wamaitha et al., 2018; Mwatuni et al., 2020). Synergistic interactions with wheat streak mosaic virus, maize dwarf mosaic virus, or sugarcane mosaic virus can result in 3.3–11.2-fold higher levels of virions (Scheets, 1998; Adams et al., 2013; Xia et al., 2016). The economic losses for maize in a heavily affected region can reach up to 100% (Rao et al., 2010; Hilker et al., 2017).

The icosahedral virion of MCMV is 30 nm in diameter with 180 copies of chemically identical coat protein (CP). The viral genomic RNA (gRNA, 4,436 or 4,437 nt) produces two sub-genomic RNA (sgRNA) in host cells, has a capped structure at the 5′ end and lacks a poly(A) tail at the 3′ end. It comprises seven open reading frames (ORFs), encoding p7a, p7b, p25, p32, p50, p111, and p31 (Nutter et al., 1989; Scheets, 2000; Stenger and French, 2008). Protein p32 is directly translated from the gRNA, although the start codon sequences are not the optimal sequences for plant ribosomes. P50 is located downstream of p32 in the genome but is only 19 bases away from the start codon of p32. The termination codon of p50 is read through to synthesize p111. Proteins p7a, p7b, p31, and p25 are synthesized by the generated sgRNA (Nutter et al., 1989; Lommel et al., 1991). Among the encoded proteins, p50 and p111 function in MCMV replication in plant protoplasts. The absence of p32 results in a two-thirds decrease of transcriptional capacity compared with the wild type; p31 participates in viral systemic infection of the plant host. Coat protein p25 cooperates with two movement proteins (MPs), p7a and p7b, in the intercellular movement of the virus in the host (Scheets, 2016).

Increasing evidence has demonstrated that the CP of plant viruses has vital multifunctional roles in viral RNA-binding activity, systemic movement, host symptom formation, gene silencing suppression, and induction of the host hypersensitive response (Azevedo et al., 2010; Park et al., 2013; Kang et al., 2015; Rehman et al., 2019). The CP of another member of the family Tombusviridae, olive latent virus 1 (OLV-1), loses the ability to systemically infect if the 49 amino acids (aa) of the C-terminal region are replaced with 39 aa (Pantaleo et al., 2006). The N-terminal region of the CP of the beet black scorch virus is essential for nuclear localization, binding of viral RNA, virion assembly, and systemic infection (Zhang et al., 2011, 2013). The CP of *turnip crinkle virus* competitively combines with AGO1 in plant cells and reduces the ability of AGO to bind siRNA and miRNA, leading to a decrease in defensive RNA silencing by the host, thus promoting virus infection (Jin and Zhu, 2010).

Although the functions of other MCMV proteins have been studied intensively and significant functional diversity has been shown for MCMV CP, p25 has not been characterized in depth. Thus, our main objectives were to (1) perform bioinformatic analysis of p25; (2) identify the function of p25 in symptom development; (3) analyze the nuclear localization signal (NLS) of p25 and its role in viral pathogenicity; (4) explore the C domain of p25 in viral movement; and (5) study the host factors participated in TMV-p25 infection.

## Materials and methods

### *In silico* analysis of the maize chlorotic mottle virus p25 protein

The nucleic acid and aa sequences of MCMV Yunnan2 p25 were downloaded from the NCBI database^1^ (JQ943666.1; AFM31229.1). The nucleic acid sequences of MCMV p25 isolated in different parts of the world were also obtained from the NCBI non-redundant database. The NLS of the MCMV p25 protein was predicted by PredictNLS^2^. Transmembrane helices were analyzed with TMHMM-2.0^3^. The putative three-dimensional structure of p25 was analyzed using the SWISS-MODEL tool^4^. A phylogenetic tree was constructed by MEGA5.22 software with the neighbor-joining method (Su et al., 2018).

### Construction of plasmids

The MCMV *p25* gene was cloned from an MCMV infectious clone vector using PCR and primer pair tobacco mosaic virus (TMV)-p25F/TMV-p25R. The amplified fragments were digested with restriction enzymes *Eco*R V and *Xho* I and inserted into the *Eco*R V/*Xho* I-digested vector pTMVcpGFP to generate pTMV-p25 (Lewandowski and Adkins, 2005). Deletion mutant HA tag-infused plasmids pTMV-p25N, pTMV-p25C1, pTMV-p25C2, pTMV-p25-HA, pTMV-p25N-HA, and pTMV-p32 were constructed with the same strategy.

For subcellular localization assays in *Nicotiana benthamiana*, the coding sequences of P25/P25N were amplified and inserted into *Xba* I/*Bam*H I sites in pYBA1152, thus forming pP25-GFP and pP25N-GFP, respectively.

To construct TRV-virus-induced gene silencing (VIGS) vectors, the partial coding sequence of *NbNPR*1, *NbCOI*1, or *NbHSP*90 was ligated into *Eco*R I/*Sma* I-treated pTRV2 to generate the recombinant plasmids pTRV2-NPR1, pTRV2-COI1, and pTRV2-HSP90, respectively.

The primers are listed in Supplementary Table 1.

### Plant growth conditions and inoculation

Wild type and TMV MP-transgenic *N. benthamiana* plants were grown in growth chambers (25°C day/23°C night, 16 h light/8 h dark). At the 4–6-leaf stage, leaves of the TMV MP-transgenic *N. benthamiana* plants were rub-inoculated with infectious RNA, as previously described (Dawson et al., 1986). TRV VIGS analysis was performed as described previously (Tang et al., 2010). The brief steps are as follows: The *Agrobacterium* GV3101 harboring pTRV1, pTRV2, and their derivatives were cultivated overnight and resuspended in agroinfiltration buffer (10 mm MES, 10 mm MgCl_2_ and 100 μM acetosyringone). After incubation at 28°C for 3–5 h, the *Agrobacterium* harboring TRV RNA1 and RNA2 (their derivative) were mixed at 1:1 ratio and infiltrated into *N. benthamiana* leaves. All the experiment was repeated three times, six plants per treatment were used for every experiment. Confocal laser scanning microscopy and transmission electron microscopy analysis.

Agroinfiltrated leaf tissues were imaged using an LSM700 confocal laser scanning microscope (Zeiss, Oberkochen, Germany). GFP was excited at 488 nm, and the emissions were captured between 497 and 510 nm. The dsRed signal was excited at 557 nm, and emissions were captured from 566 to 600 nm. Images were captured digitally and analyzed using ZEN 2010 software (Zeiss, Oberkochen, Germany).

Inoculated leaves were harvested and cut into 1 mm × 1 mm sections and then fixed in 2.5% v/v glutaraldehyde and 1% w/v osmium tetroxide sequentially. The fixed tissues were dehydrated using an ethanol series and embedded in Lowicryl K4M resin as instructed by the manufacturer (Electron Microscopy Sciences, Fort Washington, PA, United States). Ultrathin sections were cut and examined using a Hitachi-7650 TEM (Tokyo, Japan).

### RT-polymerase chain reaction and RT-qPCR

Total RNA from *N. benthamiana* plants was extracted using the RNeasy Plant Mini Kit (Qiagen, Valencia, CA, United States). Immediately, RNA was equalized, and 1 μg was used in each 20 μL reverse transcription reaction using the PrimeScript™ RT Reagent Kit (TaKaRa, Kusatsu, Japan) as instructed by the manufacturer. A total of 500 ng of each cDNA sample served as a template in the polymerase chain reaction (PCR) assay using specific primers. As a control for equal loading, primers specific to the endogenous gene *Nbactin* were used. qPCR was performed using a 2 × SYBR Premix Ex Taq™ (TaKaRa, Kusatsu, Japan) following the manufacturer’s instructions. Each construct was evaluated in three independent experiments. The primers used in RT-PCR are listed in Supplementary Table 1.

### Protein extraction and western blot

Protein extraction and western blotting were carried out as described previously (Zhan et al., 2021). Approximately 0.1 g of leaf tissue from the inoculated plants was ground in 0.3 mL protein extraction buffer (6 M urea, 1 mm EDTA, 50 mm, 124 Tris-HCl, 1% SDS, pH 7.5). The crude extracts were boiled for 5 min, then centrifuged at 12,000 × g for 10 min, and the resulting supernatant (12 μL per sample plus 3 μL 5 × loading buffer) was boiled for 5 min, then chilled on ice for 5 min. Protein samples were separated in 12.5% SDS-PAGE gels and transferred to nitrocellulose membranes. The membranes were then probed with the MCMV-p25, HA or GFP monoclonal antibody, followed by a secondary antibody conjugated to AP (Abcam, Cambridge, MA, United States). The experiment was repeated three times.

## Results

### Bioinformatic analysis of p25

**FIGURE 1:**
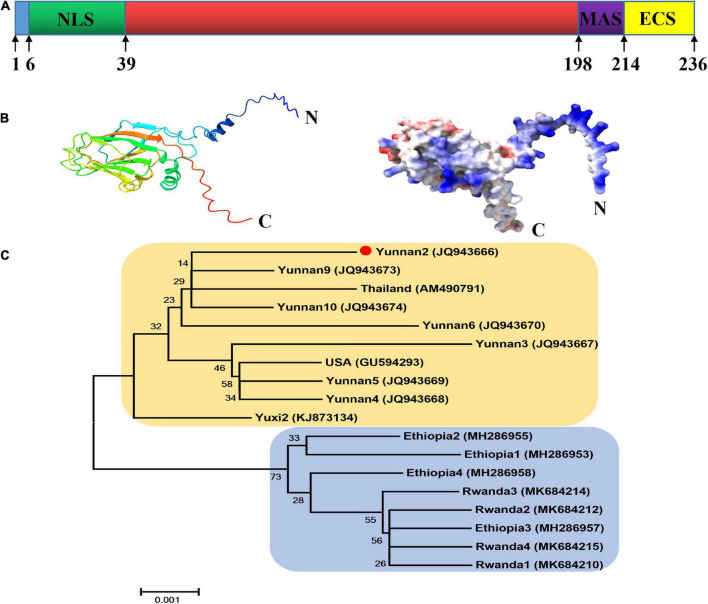
Bioinformatic analysis of the p25 of maize chlorotic mottle virus (MCMV). **(A)** Domain arrangement of p25. NLS, nuclear localization sequence; MAS, membrane-anchored sequence; ECS, extracellular sequence. Special function domains are represented with different colors. **(B)** Structural model analysis using SWISS-MODEL software. **(C)** Phylogenetic tree of CP nucleotide sequences in MCMV. The phylogenetic tree was constructed with MEGA5.22 software using the neighbor-joining method.

As shown in Figure 1A, the CP of MCMV, p25, is a 236 aa protein and contains the viral coat domain (20–204). According to the cNLS Mapper prediction analysis, position 6–38 is a putative NLS sequences. In the C-terminal of p25, aa 198–213 and 214–236 were predicted to be the membrane-anchored sequence and the extracellular sequence, respectively. The structural model analysis showed that p25 consists of 8 alpha helices and 11 beta folds (Figure 1B). In the phylogenetic analysis, to better understand the relationships among the nucleotide sequences of MCMV CP worldwide (Figure 1C), MCMV CP clustered into two distinct branches, which was further divided into three sub-branches. In China, where MCMV is mainly distributed in Yunnan Province, the sequences had high identity with that of the Thailand isolate in this study. Thus, MCMV are highly conserved worldwide.

### P25 contributes to viral pathogenicity and tobacco mosaic virus hybrid systemic movement

**FIGURE 2:**
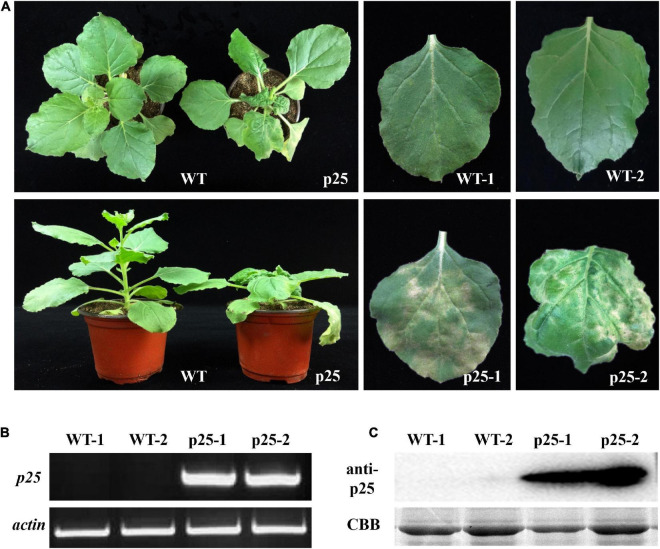
Maize chlorotic mottle virus (MCMV) p25 is required for viral pathogenicity and systemic movement in TMV MP-transgenic plants of *Nicotiana benthamiana*. **(A)** Phenotypes of transgenic plants after inoculation with TMV-p25 and TMVcp-GFP (WT) at 15 days post-inoculation. **(B)** RT-PCR analysis of RNA from leaves of plants after the two treatments. Actin from *N. benthamiana* was used as an internal standard. **(C)** Western blot detection of p25 from the leaves of plants in the two treatments. An anti-p25 antibody was used. Coomassie brilliant blue-stained Rubisco protein (CBB) was used as a total protein loading control. WT, TMV MP-transgenic plants inoculated with TMVcp-GFP; p25, TMV MP-transgenic plants inoculated with TMV-p25.1, inoculated leaves (WT or p25); 2, non-inoculated leaves on the same plant.

The ORF of p25 was introduced into the pTMVcp-GFP plasmid to generate the hybrid virus TMV-p25. Then, the RNA transcripts of TMV-p25 were used to inoculate TMV MP-transgenic *N. benthamiana* plants, and plants were inoculated with TMVcpGFP as a negative control (Zhang et al., 2012). Thereafter, the symptoms were observed and the transcript and expression level of p25 were further detected (Figure 2). The inoculated plants displayed severe symptoms (Figure 2A). The infected plants were severely stunted to half the height of the TMVcpGFP inoculated plants (Supplementary Figure 1). The inoculated leaves exhibited typical chlorosis and necrosis. Similarly, leaves that were systemically infected (leaves that were not inoculated; hereafter, systemic leaves) also had strong symptoms induced by this chimera virus.

To confirm that symptoms were induced by the presence of p25, we used RT-PCR and western blot analysis to detect the p25 transcript and protein levels, respectively (Figures 2B,C). Intense bands for p25 appeared for inoculated and systemic leaves but not for the TMVcpGFP-inoculated leaves. These results indicate that p25 is required for viral pathogenicity and systemic movement in *N. benthamiana*.

### Maize chlorotic mottle virus p25 is located in the nucleus

**FIGURE 3:**
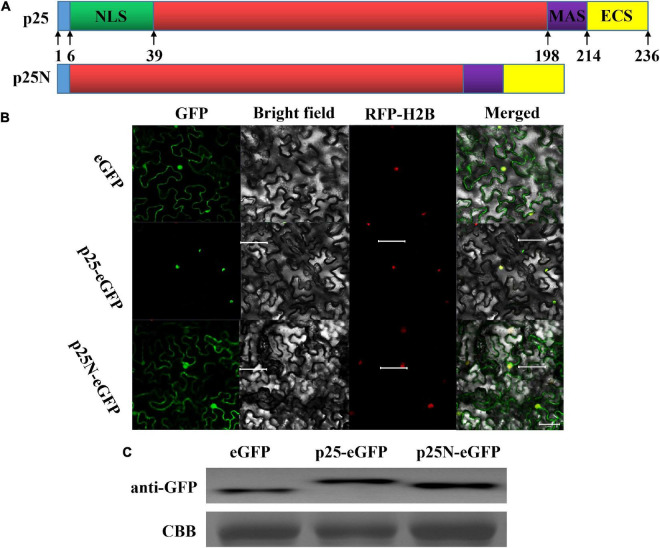
Subcellular location of p25. **(A)** Schematic sketch of the construction strategies of p25 and p25N. p25, the whole open reading frame (ORF) of p25; p25N, the truncated sequence lacking the nuclear localization signal (NLS) (in green) of p25. **(B)** Confocal laser scanning micrographs of the location of p25 in *Nicotiana benthamiana*. The co-localization signal of RFP-H2B is shown as a positive nuclear control, and eGFP was used as a control located in the nucleus and cytoplasm. Scale bars: 50 μm. **(C)** Western blot of eGFP, p25-eGFP, and p25N-eGFP using anti-GFP antibody. Coomassie brilliant blue-stained Rubisco protein (blue bands) was used as a total protein loading control.

A stretch of basic residues in the N-terminal region of MCMV p25 resembled those of NLS. Leaves of *N. benthamiana* were examined with confocal laser scanning microscopy after the ORF of p25, and p25 lacking the NLS (p25N) were inserted into vector pYBA1152 to transiently express an eGFP-fused protein, driven by the 35S promoter (Figure 3A). As shown in Figure 3B, RFP-H2B was detected in the nucleus, and eGFP was detected in the nucleus and cytoplasm. As expected, p25-eGFP was uniquely located in the nucleus, whereas p25N-eGFP was found in the nucleus and cytoplasm, which is consistent with free eGFP localization. The use of p25-eGFP and p25N-eGFP with RFP-H2B/eGFP shows that p25 is located in the nucleus, and the NLS of p25 is needed for transport and restriction to the nucleus. Moreover, the western blot result indicates that the expressions of eGFP, p25-eGFP and p25N-eGFP proteins were correct and stabilized in *N. benthamiana* leaves (Figure 3C).

### The nuclear localization signal of p25 is required for pathogenicity

**FIGURE 4:**
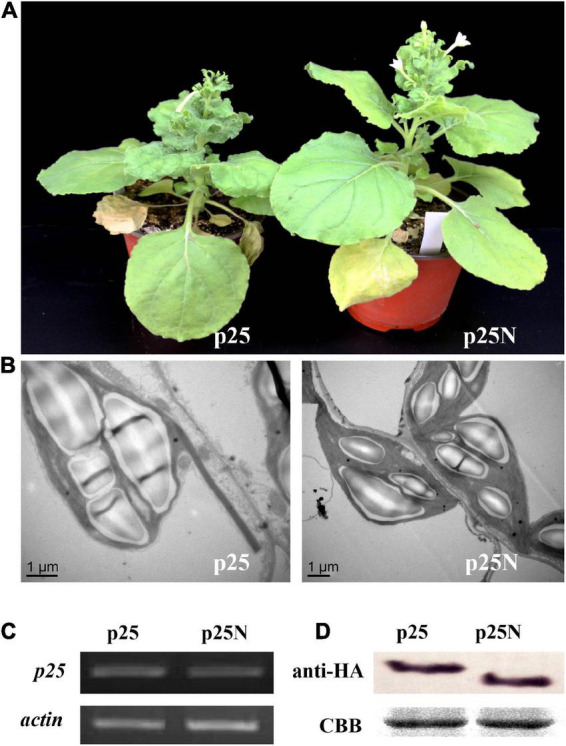
Pathogenicity analysis of the nuclear localization signal (NLS) in p25 using *Nicotiana benthamiana* plants. p25, plants inoculated with hybrid virus TMV-p25; p25N, plants inoculated with hybrid virus TMV-p25N lacking NLS. **(A)** Phenotype of plants inoculated with either the hybrid virus TMV-p25 or TMV-p25N. **(B)** Transmission electron micrographs of infected leaves. Scale bars: 1 μm. **(C)** Transcripts were detected by RT-PCR using extracted leaf RNA that was reverse transcribed. The actin of *N. benthamiana* was used as the internal control. **(D)** Western blot of p25 using an anti-HA antibody. Coomassie brilliant blue-stained Rubisco protein (blue bands) is shown as a total protein loading control.

To further determine whether the NLS of p25 is involved in pathogenicity, TMV MP-transgenic plants were inoculated with a hybrid virus, TMV-p25 or TMV-p25N, then examined for symptoms and analyzed for p25 transcript and protein levels (Figure 4). In Figure 4A, although the plants inoculated with TMV-p25N had viral symptoms, the plants were distinctly taller than those inoculated with TMV-p25 (Supplementary Figure 1). As viewed by transmission electron microscopy observation, starch grains in the chloroplasts of TMV-p25 inoculated leaves were significantly larger than in those inoculated with TMV-p25N (Figure 4B). However, the transcription and expression levels of p25 and p25N were not affected between TMV-p25 and TMV-p25N in leaves (Figures 4C,D). These results suggest that the NLS of p25 is important for full pathogenicity.

### Deletion of the C-terminal region of p25 impairs viral systemic movement and pathogenicity

**FIGURE 5:**
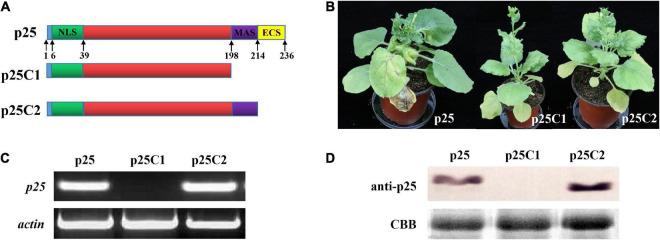
Functional analysis of the C-terminal region of p25. **(A)** The fused protein constructs. p25, plant inoculated with hybrid virus TMV-p25;. p25C1, plant inoculated with hybrid virus TMV-p25C1; p25C2, plant inoculated with hybrid virus TMV-p25C2. **(B)** Phenotype of *Nicotiana benthamiana* plants after inoculation with hybrid virus TMV-p25, TMV-p25C1, or TMV-p25C2. **(C)** The transcriptional level was detected by RT-PCR using extracted leaf RNA after reverse transcription. *N. benthamiana* actin was used as the internal control. **(D)** Western blot of p25 using anti-p25 antibody. Coomassie brilliant blue-stained Rubisco protein (blue bands) was used as a total protein loading control.

To study the relevance of the C-terminal region of p25 in viral systemic movement and pathogenicity, we deleted the key domain and used the resultant constructs to inoculate TMV MP-transgenic *N. benthamiana* plants (Figure 5). As shown in Figure 5A, we deleted the 198–236 or 214–236 domain in the C-terminal region of p25 and inserted the respective constructs into the pTMVcpGFP plasmid to generate hybrid viruses TMV-p25C1 and TMV-p25C2. Plants inoculated with TMV-p25C1 developed mild symptoms and were taller than those inoculated with TMV-p25 or TMV-p25C2 (Supplementary Figure 1); plants inoculated with TMV-p25 or TMV-p25C2 exhibited very similar symptoms (Figure 5B). Similarly, p25 and p25C2 mRNA transcripts and protein were detected in systemic leaves but not in TMV-p25C1 inoculated leaves, which were defective for system infection (Figures 5C,D). These data demonstrate that the 198–236 residues in the C-terminal of p25 are not only involved in viral systemic movement but are also required for pathogenicity.

### *Nicotiana benthamiana HSP90* involved in p25 mediated viral pathogenicity

**FIGURE 6:**
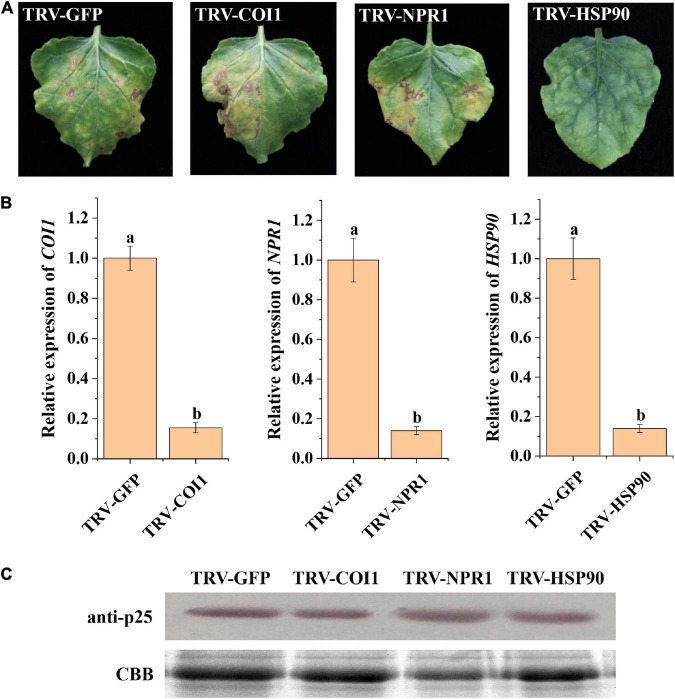
Analysis of plant resistance genes involved in TMV-p25 infection. **(A)** Leaves were infiltrated with *Agrobacterium*-containing pTRV1 or pTRV2-NbNPR1/NbCOI1/NbHSP90 plasmids. pTRV2-GFP was used as a control, and then plants were inoculated with hybrid virus TMV-p25. **(B)** RT-qPCR analysis of *Nicotiana benthamiana* leaves after VIGS. Error bars represent standard deviations. Different letters above the bars for each treatment indicate a significant difference (Student’s *t*-test, *p* < 0.05). **(C)** Western blot of p25 protein using the anti-p25 antibody. The Rubisco protein was stained with Coomassie brilliant blue (blue bands) as a total protein loading control.

Previous studies have shown that host resistance genes participate in the interactions between viruses and their host plants (Liu et al., 2004; Tran et al., 2016; Zhao et al., 2019). Thus, we selected resistance genes COI1, NPR1, and HSP90 as potential candidates interacting with p25 in TMV MP-transgenic *N. benthamiana* plants after inoculation with hybrid virus TMV-p25 (Figure 6). The leaves with silenced COI1 and NPR1 were similar to the control, whereas silenced HSP90 resulted in tissue chlorosis rather than necrosis (Figure 6A). RT-qPCR analysis revealed that *COI1*, *NPR1*, and *HSP90* were significantly downregulated by the TRV-VIGS system compared with the levels in the control (Figure 6B). Western blots showed typical p25 levels after all treatments (Figure 6C). These results suggest that host HSP90 is required for the p25 mediated viral pathogenicity and host resistance.

## Discussion

Plant viral CP has multiple functions, not only as a structural protein that can encapsidate the viral DNA/RNA to form virions, it also plays an important role in viral infection, proliferation, cell to cell movement, pathogenicity, and vector transmission (Callaway et al., 2001). Here, we found that MCMV CP- p25, consists of 236 aa and is highly conserved in MCMV isolates from around the world. Bioinformatic analysis showed that p25 contains the typical viral CP domain, including the NLS (aa 6–38), membrane-anchored sequence (aa 198–213), and extracellular sequence (aa 214–236). We demonstrated that p25 caused the severe dwarfing and necrosis and was transported systemically in *N. benthamiana* by a TMV-based expression system. Subcellular localization analysis revealed that the NLS of p25 was exclusively localized in the host nucleus. This domain was also necessary for pathogenicity. Moreover, viral systemic movement in *N. benthamiana* was significantly weakened due to the deletion of the C-terminal region of p25, especially aa 198–213. Finally, the VIGS results showed that HSP90 may involve in TMV-p25 induced tissue necrosis. However, the mechanism of symptom development is undetermined.

Using chimeric viruses is a common strategy to study gene function in plant viruses, especially in negative-strand RNA viruses and some viruses that are difficult to manipulate (Yu et al., 2019; Mei et al., 2020). Thus, p25 is an important virulence factor in the TMV-p25 chimeric virus, consistent with the role of many viral CPs. The CP of TMV is broken down into monomers when it binds with the antimicrobial agent ningnanmycin, nearly resulting in the loss of pathogenicity (Li et al., 2017). The CP of cucumber mosaic virus (CMV) is also a determinant of chlorotic symptoms in infected hosts using chimeric ICs (Qiu et al., 2018). We also generated an NLS deletion mutant to examine subcellular localization and pathogenicity in plants and found that p25 is exclusively localized in the nucleus. At the N-terminal, the NLS is rich in basic aa that are essential for the nuclear location, consistent with the findings of a previous study (Zhan et al., 2016). However, Zhan et al. (2016) did not find that the p25 NLS was involved in pathogenicity. Here, we found that the deletion mutant protein did not affect viral accumulation but resulted in almost a total loss of pathogenicity. Growing evidence suggests that the location of the viral CP is responsible for symptom expression in the host (Liu et al., 2009). The N-terminal lysine-rich fragment induces the alfalfa mosaic virus CP to localize to the nucleus and accumulate in the nucleolus of infected plant cells (Herranz et al., 2012). The localization of pepper mild mottle virus changes from the chloroplast to the cell periphery when Asp is substituted for Asn at one location in the CP, which induces different symptoms (Han et al., 2020). The C-terminal region in viral CP has also been shown to be involved in viral cell-to-cell transport in plants (Fedorkin et al., 2001). In our current study, p25 mRNA transcripts or proteins were not detected in systemic leaves when the C-terminal was deleted from p25, which agrees with the significant reduction in symptom severity, including dwarfing and necrosis, in the whole plant. Thus, we consider C-terminal p25 to be indispensable for the long-distance movement and pathogenicity of the TMV-p25 chimeric virus. These findings encouraged us to further consider the subcellular localization of p25 and which domain plays a decisive role in pathogenicity.

Heat shock protein 90 (HSP90), a highly conserved protein in eukaryotes, participates in stimulating signal proteins, including protein kinases, hormone receptors, and transcription factors (Pearl and Prodromou, 2006). HSP90 also play an important role in the ubiquitination processes of defense response regulators in plants (Ishii et al., 2008; Chen et al., 2010; Kadota and Shirasu, 2012). HSP90-silenced plants do not develop the hypersensitive response (HR) elicited by INF1 and *Pseudomonas cichorii*, and the expression levels of pathogenesis-related protein genes decreased (Kanzaki et al., 2003). The activation of kinases NtMEK2, WIPK, and SIPK are also significantly inhibited when HSP90 is suppressed (Takabatake et al., 2007). In our study, necrosis did not develop in HSP90-silenced leaves, and p25 levels were almost the same in the different treatments. These results indicated that HSP90 is important for symptom development in the host plant rather than facilitating infection and replication, providing clues about the molecular mechanism of HSP90 essential in the pathway elicited by TMV-p25.

Other studies have suggested that CP interacts strongly with factors in host plants and other organisms. The long-distance movement of tomato mosaic virus is closely related to the interaction between viral CP and IP-L, which is located in the thylakoid membranes of tobacco (Zhang et al., 2008). Using the mutation of aa residues and a yeast two-hybrid system, soybean mosaic virus (SMV) CP was shown to interact with helper-component proteinase to enable the virus to be transmitted to plants by aphids (Seo et al., 2010). Moreover, the SMV CP interacts with soybean GmCPIP to facilitate viral infection and accumulation in soybean (Zong et al., 2020). Cucumber mosaic virus CP acts as a bridge between viral-encoded suppressors and host RNA silencing to facilitate viral self-attenuation and symptom recovery (Zhang et al., 2017). Resultantly, we considered that CP interactions with a host factor(s) were involved in viral invasion, symptom formation, and systemic movement; future studies will aim to determine more host factors by using a yeast two-hybrid system and bimolecular fluorescence complementation to better understand the involvement of MCMV p25 in symptom development and long-distance transport.

## Data availability statement

The original contributions presented in this study are included in the article/Supplementary material, further inquiries can be directed to the corresponding authors.

## Author contributions

XS and JL: conceptualization. WL: methodology and software. XS: validation. CZ and DW: formal analysis, data curation, and writing—original draft preparation. CZ, BZ, GA-F, and DW: investigation. CZ: resources and funding acquisition. XS and CZ: writing—review and editing. JL: visualization and supervision. All authors have read and agreed to the published version of the manuscript.

## Footnotes

1
https://www.ncbi.nlm.nih.gov/

2
https://rostlab.org/owiki/index.php/PredictNLS

3
https://services.healthtech.dtu.dk/service.php?TMHMM-2.0

4
http://www.swissmodel.expasy.org
