# Supplementary material for: The coat protein p25 from maize chlorotic mottle virus involved in symptom development and systemic movement of tobacco mosaic virus hybrids
Source: Front Microbiol. 2022 Aug 5;13:951479. doi: 10.3389/fmicb.2022.951479 (PMC9389212; doi:10.3389/fmicb.2022.951479)
Supplement: Supplementary file 1 [file Image_1.pdf]

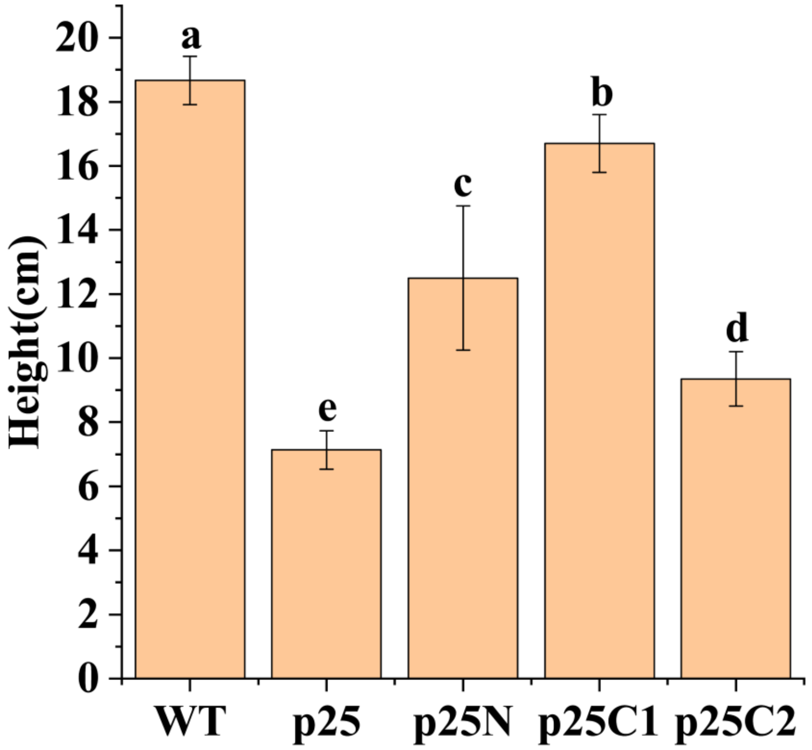

Figure S1 Height statistical analysis of plants inoculated by TMV-p25, TMV-p25N, TMV-p25C1 and TMV-p25C2 15 days post inoculation, respectively .
