# Supplementary material for: The coat protein p25 from maize chlorotic mottle virus involved in symptom development and systemic movement of tobacco mosaic virus hybrids
Source: Front Microbiol. 2022 Aug 5;13:951479. doi: 10.3389/fmicb.2022.951479 (PMC9389212; doi:10.3389/fmicb.2022.951479)
Supplement: Supplementary file 2 [file Table_1.DOCX]

Supplementary Material

Table S1. The primers used in this study^1^

| **Construct name** | **Primer name** | **Primer sequence (5＇-3＇)** |
| --- | --- | --- |
| pTMV-p25  pTMV-p25N-  pP25-GFP  Pp25NLS-GFP  pTMVp25C1  pTMVp25C2  pTMVp25-HA  pTMVp25N-HA  pTRV2-COI1  pTRV2-NPR1  pTRV2-HSP90  RT-qPCR | TMV-p25-F  TMV-p25-R  TMV-p25N-F  TMV-p25-R  p25gfp-F  p25gfp-R  p25Ngfp-F  p25gfp-R  TMV-p25-F  TMV-p25C1-R  TMV-p25-F  TMV-p25C2-R  TMV-p25-F  TMV-p25HA-R  TMV-p25N-F  TMV-p25HA-R  NbCOI1-F  NbCOI1-R  NbNPR1-F  NbNPR1-R  NbHSP90-F  NbHSP90-R  OligodT  Nbactin-F  Nbactin-R  NbCOI1-F  NbCOI1-R  NbNPR1-F  NbNPR1-R  NbHSP90-F  NbHSP90-R | TA**GATATC**ATGGCGGCAAGTAGCCGG  AA**CTCGAG**TCAATGATTTGCCAGCCCT  TA**GATATC**ATGGCGGCAAGTAGCCGGCCCGCAGCGAAACCGTCC  AA**CTCGAG**TCAATGATTTGCCAGCCCT  TA**TCTAGA**ATGGCGGCAAGTAGCCGG  AA**GGATCC**ATGATTTGCCAGCCCTGGGC  TA**TCTAGA**ATGGCGGCAAGTAGCCGGCC  AA**GGATCC**ATGATTTGCCAGCCCTGGGC  TA**GATATC**ATGGCGGCAAGTAGCCGG  AC**CTCGAG**TCAGATAGCCACAATGAATC  TA**GATATC**ATGGCGGCAAGTAGCCGG  AC**CTCGAG**TCAGTACGAG ATTTTGATTT  TA**GATATC**ATGGCGGCAAGTAGCCGG  AC**CTCGAG**TCAGGCATAATCCGGCACATCATAAGGGTAATGATTTGCCAGCCCT  TA**GATATC**ATGGCGGCAAGTAGCCGGCCCGCAGCGAAACCGTCC  AC**CTCGAG**TCAGGCATAATCCGGCACATCATAAGGGTAATGATTTGCCAGCCCT  ag**GAATTC**AGCAGCCCATTGTTTCTTAC  at**CCCGGG**CGGACACCATTATCAAGTGG  ag**GAATTC**AGTTTGACTACTTCGCCGAC  at**CCCGGG**ACCTCAGCAAGGAACGCTA  ag**GAATTC**TGAGCCCAAGAAAGACGA  at**CCCGGG**CCATAATGAACACCCTCCTG  TTTTTTTTTTTTTTTTTT  GCAGGAATCCACGAGACTACA  AACCTCCAATCCAGACACTGT  GCTTAGAAGGTTTGCCCTC  CTGAAGATGCCCTGTATCCT  TGTATCTCTTGCTATGGCAGG  TCTACCGTTGTCCTCTGTGC  GTGTTGTTGCTCTTTGAGACAG  TCCATCTTGCTTTCTTCACC |

1. The restriction recognition sequences of *Eco*RⅤ (GATATC), *Xho*Ⅰ (CTCGAG), *Xba*Ⅰ (TCTAGA), *Bam*HI (GGATCC), *Eco*RⅠ(GAATTC) and *Sma*Ⅰ (CCCGGG) within the primers are in bold and underlined. Genes included in qPCR assay are *Nbactin* (GenBank No. JQ256516.1) and *NbCOI1* (Niben101Scf02280g08005.1), *NbNPR1* (Niben101Scf14780g01001.1), *NbHSP90* (Niben101Scf01475g00019.1), from *Nicotiana benthamiana* draft genome sequence V1.0.1 (https://solgenomics.net/organism/Nicotiana_benthamiana/genome).
